# The anatomy and histology of the midgut and Malpighian tubules of *Copris felschei* Reitter, 1892 (Coleoptera: Scarabaeidae)

**DOI:** 10.1007/s00709-024-02021-1

**Published:** 2025-01-11

**Authors:** Nurcan Özyurt Koçakoğlu, Doğan Erhan Ersoy, Hicret Arslan, Selami Candan

**Affiliations:** Department of Biology, Faculty of Science, Gazi University, Ankara, Turkey

**Keywords:** Dung beetle, Midgut, Malpighian tubules, Light microscopy, Electron microscopy

## Abstract

*Copris* are part of the Scarabaeidae family of Coleoptera. *Copris* are dung beetles or coprophagous beetles. These insects are called tunnelers because they excavate channels in the substrate. They use dead organisms and non-living organic compounds as a nutrient source. By breaking down dead matter, they provide nutrients that are important to the environment and necessary for the survival of other organisms. No studies have yet examined the midgut structure and Malpighian tubules of *Copris*. Therefore, this study investigated the histo-anatomical structure of the midgut and Malpighian tubules of *Copris felschei* Reitter, 1892 (Coleoptera: Scarabaeidae) using light and scanning electron microscopy (SEM) in detail. The midgut of *C. felschei* represents the largest part of the alimentary canal. Muscle layers and a monolayer of cylindrical epithelium surround the midgut wall. A peritrophic membrane envelops food in the midgut lumen, and crystals were observed within the lumen. The surface of the midgut has regenerative crypts and tracheae. The Malpighian tubules are arranged in two pairs and connect proximally between the midgut and hindgut. The Malpighian tubules are composed of a single layer of cuboidal epithelium. Numerous balloon-like tracheae were observed interspersed between the midgut and Malpighian tubules. Light and SEM images of the tracheae reveal a spongy structure with hollow chambers. These findings are anticipated to advance future research and deepen our understanding of the alimentary canal in Coleoptera, particularly within the Scarabaeidae family.

## Introduction

Scarabaeoidea represents a large Coleoptera group with approximately 35,000 species distributed worldwide (Grebennikov and Scholtz 2004). The Scarabaeidae family encompasses 6,762 species and subspecies from 521 genera in the Palearctic region. In Turkey, there are 524 species and subspecies from 115 genera. The genus *Copris* is represented by 55 species in the Palearctic (Löbl and Daniel 2016). Among these, the species *Copris armeniacus* Faldermann, 1835, *C. hispanus* *cavolinii* V. Petagna, 1792, *C. hispanus hispanus* Linnaeus, 1764, *C. lunaris* Linnaeus, 1758, and *C. umbilicatus* Abeille de Perrin, 1901 are found in Turkey (Löbl and Daniel 2016; Ziani 2017). Dung beetles (Coleoptera: Scarabaeidae) are predominantly coprophagous insects, specializing in consuming fresh manure as their primary source of nutrition. These insects are called “tunnelers” because they excavate canals within the dung to facilitate feeding (Scholtz et al. 2009). In addition to coprophagy, dung beetles may also utilize other organic materials, such as carrion, fruit, and fungi, for nourishment; some species are even predatory toward other invertebrates (Noriega and Calle 2008; Halffter and Halffter 2009; Scholtz et al. 2009). Scarabaeine plays crucial ecological roles within ecosystems. By using mammalian dung for both feeding and reproduction, dung beetles provide a range of ecosystem services, including fecal clean-up, nutrient recycling, soil fertilization, greenhouse gas mitigation, secondary seed dispersal, pest control, and parasite suppression (Nichols et al. 2008; Scholtz et al. 2009; Raine and Slade 2019).

The alimentary canal, one of the most distinctive internal organs of the Coleoptera, exhibits considerable structural diversity that correlates with the different feeding strategies employed by beetle species (Özyurt Koçakoğlu et al. 2021a, 2022a). However, some perspectives suggest that the characteristics of the intestine may be more closely related to the phylogeny of the alimentary canal rather than feeding habits (Mehrabadi et al. 2012; Toni et al. 2022). Like other insects, the alimentary canal of Coleoptera (beetles) is a large tubular structure that occupies a significant portion of the insect’s body (Wigglesworth 1972). The alimentary canal of insects is a continuous, straight, or curved tube extending from the mouth to the anus (Crome 1957).

The alimentary canal is generally divided into three regions: the foregut of ectodermal origin, the midgut of endodermal origin, and the hindgut, also of ectodermal origin (Sinha 1958; Snodgrass 1993; Rubio et al. 2008; Aldiagil et al. 2013; De Sousa et al. 2013). These regions are involved in food intake, storage, digestion, absorption, and maintaining water balance (Borror et al. 1976; Calder 1989; Romoser and Stoffolano 1998). The foregut comprises the pharynx, esophagus, crop, and proventriculus (Aslam 1961; Crowson 1981). The foregut functions primarily as a mechanical filtration unit and sometimes as a temporary food storage site (Jaspar-Versali et al. 1987). The midgut is the main site for food digestion and nutrient absorption (Johnson and Rabosky 2000), and it is particularly crucial for maintaining ion balance and water transport (Dow 1986; Terra 1990). The surface of the midgut may be smooth and uniform, or it may exhibit spherical, filiform, elongated, or finger-like papillae, with variations in location, number, arrangement, and nomenclature among different species and groups (Thomas 1967; Crowson 1981; Calder 1989; Candan et al. 2020a; Özyurt Koçakoğlu et al. 2020a, 2021b, 2022a; Toni et al. 2022). The hindgut is divided into three parts: the ileum, colon, and rectum (Bess 1935). It plays a critical role in the temporary storage and excretion of food residues and the reabsorption of water and minerals from dung before excretion (Ekis and Gupta 1971; Gillott 2005).

Malpighian tubules are important components of the excretory system and develop from the ectoderm (Stammer 1934). Most Coleoptera species have either four or six Malpighian tubules. The openings of the proximal part of Malpighian tubules are located at the junction between the midgut and the hindgut. Distal part either wander within the hemocoelic cavity or form a cryptonephridial system with the hindgut (Poll 1932; Crowson 1981; Saini 2009; Yang et al. 2009, 2011; Song et al. 2012; Candan et al. 2020b, 2020a; Özyurt Koçakoğlu 2022a).

The alimentary canal of insects has a relatively complex structure that varies from species to species. Therefore, in this study, the anatomy and histology of the midgut and Malpighian tubules of *C. felschei* (Scarabaeidae), a species whose alimentary canal has not been previously studied and is of significant ecological importance, is described and presents its similarities and differences with other beetles. This is the first report on histo-anatomy of midgut and Malpighian tubules in *Copris*. The anatomical and histological studies of the alimentary canal are important for comprehending the basic organization. We aim to contribute to future research and enhance our understanding of the alimentary canal in Coleoptera, including Scarabaeidae.

## Material and methods

### Insect and stereomicroscope (SM)

In April 2024, adult specimens (male and female) of the sampled *C. felschei* were collected from Ballıkaya (40.941379°, 35.575285°), Amasya, Turkey. This sample was collected with E-21264211-288.04−11114012 numbered research permission of T.C. Ministry of Agriculture and Forestry by Doğan Erhan Ersoy. The insects were first anesthetized using ethyl acetate fumes, then dissected in 0.1 M sodium phosphate buffer (pH 7.2), and subsequently examined and photographed using an Olympus SZX7 stereomicroscope (SM).

### Light microscope (LM)

For histological observations, specimens were first dissected, and the midgut and Malpighian tubules were fixed in 10% neutral formalin for 24 h. The samples were then washed in tap water, followed by dehydration through a graded ethanol series from 50% to 99%. Subsequently, the samples were cleared in two 15-min xylol series. The tissues were then progressively transitioned from xylol to paraffin. The samples were embedded in liquid paraffin at 65 °C, and the paraffin was allowed to solidify at room temperature. Sections of 5–6-μm thickness were cut from the paraffin blocks using a Microm HM 310 microtome. These sections were stained with hematoxylin-eosin and Mallory’s triple stain and analyzed using an Olympus BX51 light microscope (LM).

### Scanning electron microscope (SEM)

For scanning electron microscopy (SEM) examinations, the midgut and Malpighian tubules were initially fixed in 2.5% glutaraldehyde and washed with sodium phosphate buffer (pH 7.2). The samples underwent a graded dehydration series in ethanol, progressing from 50% to 99%. Following dehydration, the samples were treated with hexamethyldisilazane (HMDS) and allowed to air dry. The samples were photographed intact, then deliberately broken in specific areas, mounted on stubs with double-sided adhesive tape, and coated with a thin gold layer under vacuum using a Polaron SC502 coating device. The SEM analyses were conducted with a JEOL JSM 6060 scanning electron microscope (SEM) at Gazi University, and the photographs were taken at 5 kV.

## Result

**Fig. 1, 2:**
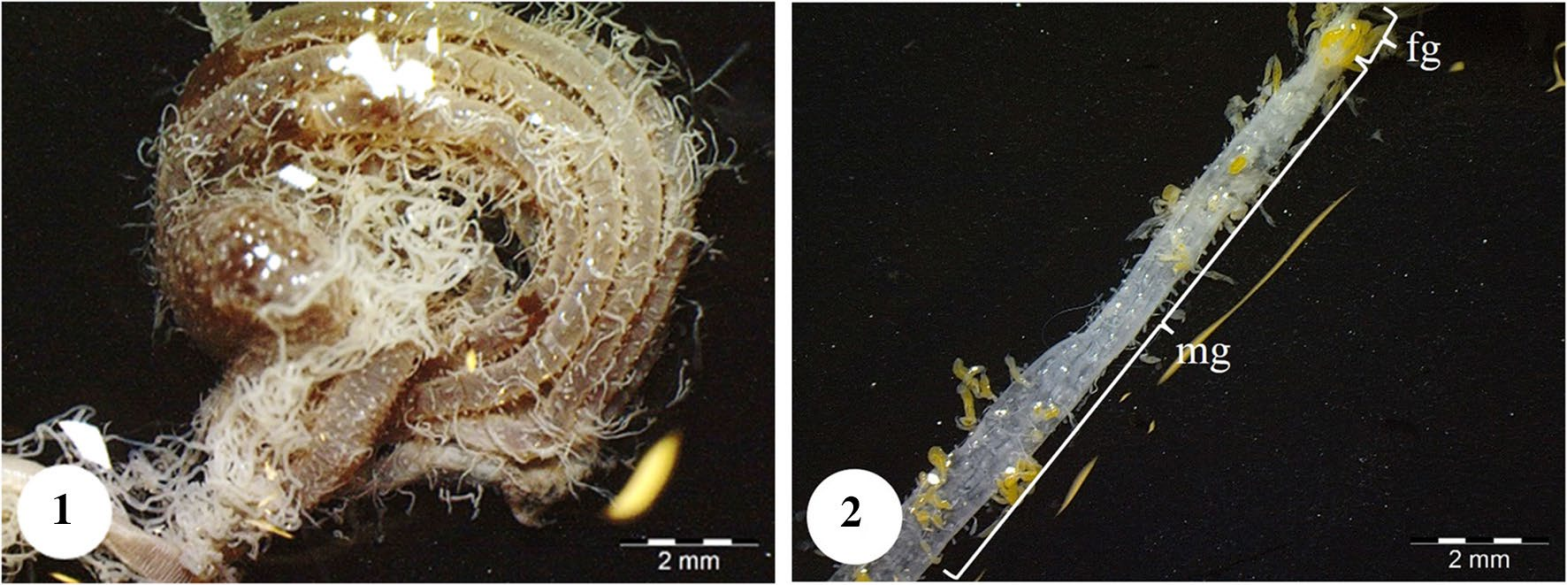
SM photo of five to six annular wrapped midguts. **2**. General view of foregut and midgut. *fg* Foregut, *mg* midgut, *SM* stereomicroscope

**Fig. 3, 4:**
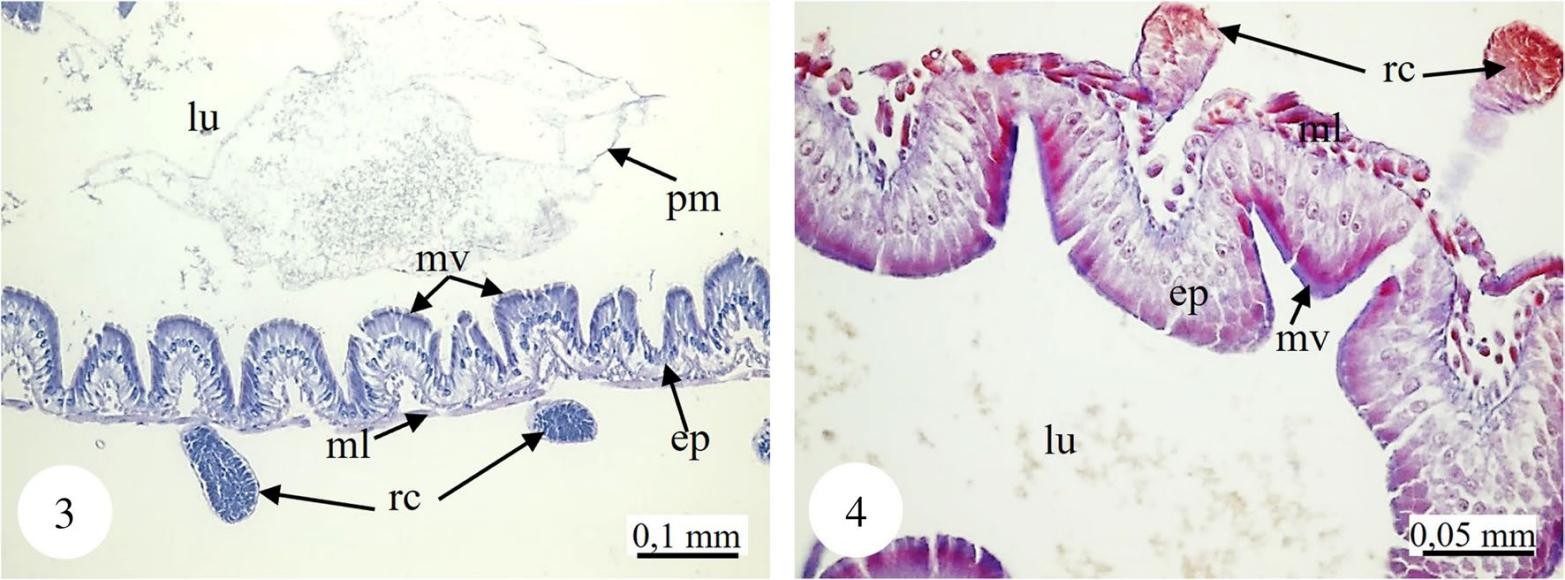
Histological section of the regenerative crypt and, the monolayered cylindrical epithelium and muscle surrounding the midgut (H&E, M), (LM). *ep* Epithelium, *lu* lumen, *ml* muscle layer, *mv* microvilli, *rc* regenerative crypt, *pm* peritrophic membrane, *H&E* hematoxylin-eosin, *M* Mallory’s triple stain, *LM* light microscope

**Fig. 5−8:**
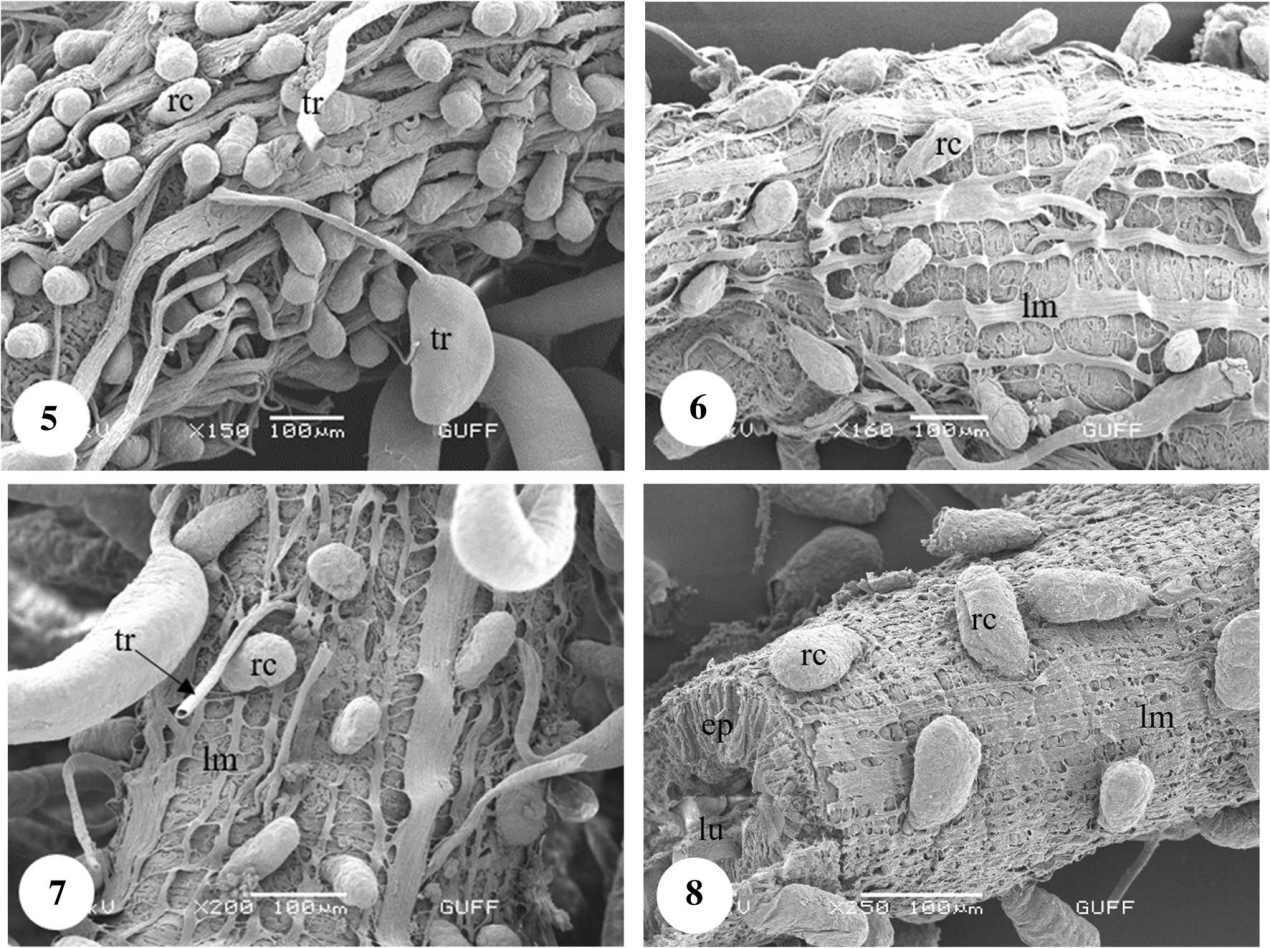
SEM photographs of trachea, longitudinal muscle and regenerative crypts on the midgut surface. *lu* Lumen, *rc* regenerative crypt, *lm* longitudinal muscle, *tr* trachea, *ep* epithelium, *SEM* scanning electron microscope

**Fig. 9−11:**
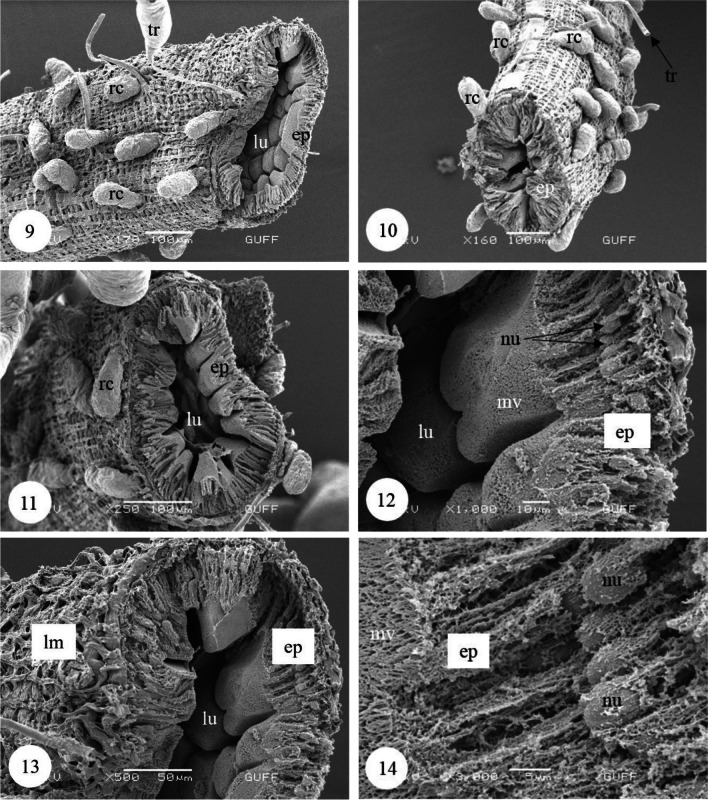
SEM photographs of the monolayered cylindrical epithelium and muscle layer surrounding the midgut, and the regenerative crypts located on the surface of the midgut. **12–14**. Detailed view of the epithelium with oval nuclei and microvilli surrounding the midgut (SEM). *lu* Lumen, *rc* regenerative crypt, *tr* trachea, *ep* epithelium, *lm* longitudinal muscles, *mv* microvilli, *nu* nucleus, *SEM* scanning electron microscope

**Fig. 15, 16:**
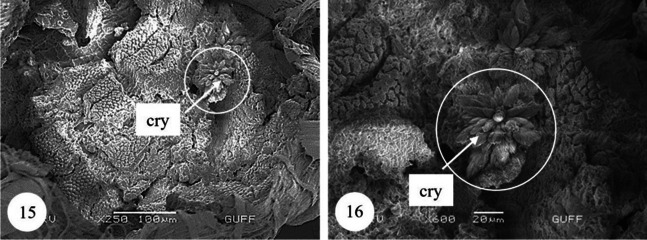
SEM photographs of crystals in the midgut lumen. *cry* Crystals, *SEM* scanning electron microscope

**Fig. 17, 18:**
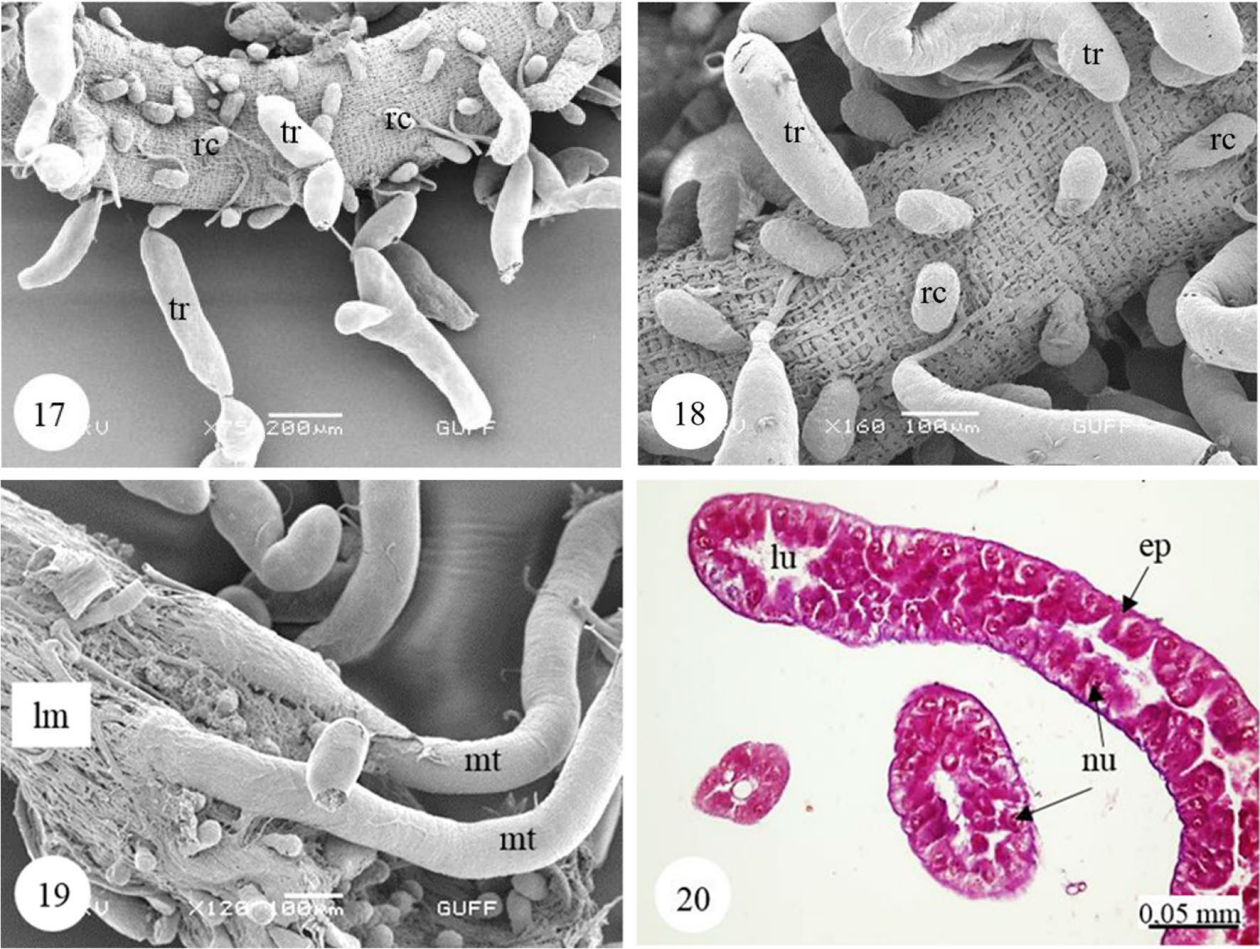
SEM photographs of many tracheae forming finger-shaped balloon-like structures between the regenerative crypts on the outer surface of the midgut. **19**. The connection between the midgut and hindgut from the proximal part of the Malpighian tubule (SEM). **20**. The transverse and longitudinal section of Malpighian tubule (M) (LM). *lu* lumen, *Mt* Malpighian tubule, *rc* regenerative crypt, *tr* trachea, *ep* epithelium, *lm* longitudinal muscles, *nu* nucleus, *M* Mallory’s triple stain, *Lm* light microscope, *SEM* scanning electron microscope

In *Copris felschei*, the alimentary canal consists of three parts: foregut, midgut, and hindgut. The longest part is the midgut, which is the main organ of digestion (Figs. 1, 2). The foregut is relatively short and continues with the midgut (Fig. 2), which has a tubular structure with regenerative crypts (Figs. 3, 4, 5, 6, 7, 8, 9, 10, 11). Long, tubular structures then form five to six annular circles with numerous tracheae (Fig. 1). In histological sections, it is distinguished that the midgut wall is surrounded by a single-layered cylindrical epithelium and muscle layer from inside to outside (Figs. 3, 4). The nuclei are oval, aligned near the basal of the epithelial cell, and are chromatin-dense (Figs. 3, 4). Epithelial cells have folds towards the lumen (Figs. 3, 4). There are microvilli (striated border) in the apical part of the epithelial cells, and absorption increases with the expansion of the apical surface of the epithelial cells (Figs. 3, 4). A peritrophic membrane surrounds the nutrients in the lumen (Fig. 3). Regenerative crypts have connections with midgut epithelial cells. The regenerative crypt has a single layer of columnar epithelium with microvilli at the apical (Figs. 3, 4). In SEM photographs of the midgut, many regenerative crypts are distinguished on the surface (Figs. 5, 6, 7, 8, 9, 10, 11). SEM photographs of broken samples of dried midgut revealed that the midgut wall is surrounded by a single layer of cylindrical epithelium and muscle layer (Figs. 9, 10, 11). Longitudinal muscles and thin connections between them are distinguished (Figs. 6, 7). Oval nuclei are distinguished near the basal of the epithelial cells, and short microvilli at the apical part are distinguished (Figs. 12, 13, 14). Crystal structures can be seen in some places in the midgut lumen (Figs. 15, 16). It is connected to the midgut wall with a thin filamentous structure from the proximal part of many trachea, forming finger-shaped balloon-like structures between the regenerative crypts on the outer surface of the midgut (Figs. 17, 18).

**Fig. 21−23:**
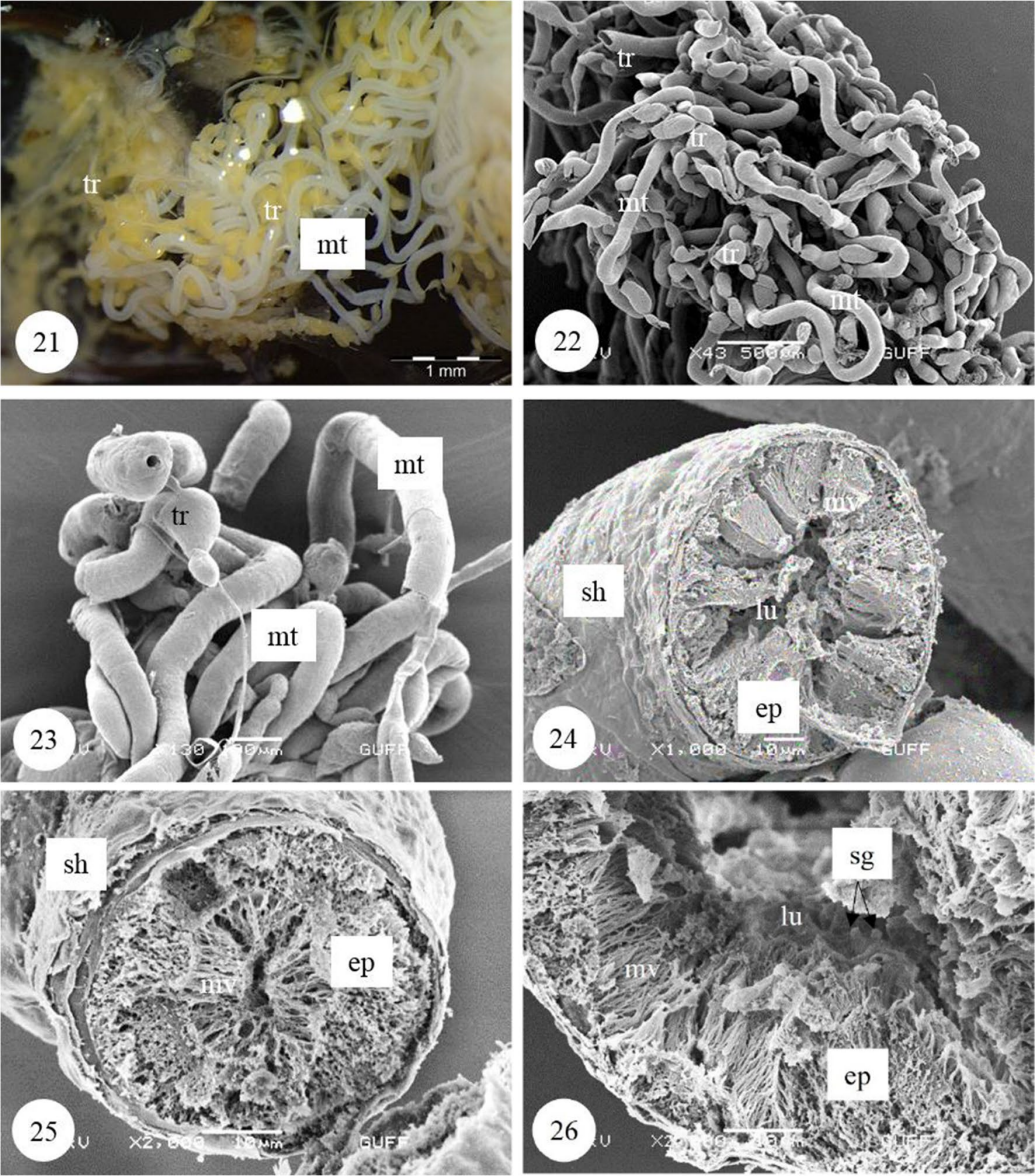
SM and SEM photographs of the Malpighian tubules and the numerous tracheae located between these tubules. **24–26**. Long microvilli extending from the apical of the single-layered cuboidal epithelium surrounding Malpighian tubule wall (SEM). *Mt* Malpighian tubule, *tr* trachea, *lu* lumen, *ep* epithelium, *mv* microvilli, *sg* secretion granules, *sh* sheath, *SM* stereomicroscope, *SEM* scanning electron microscope

**Fig. 27:**
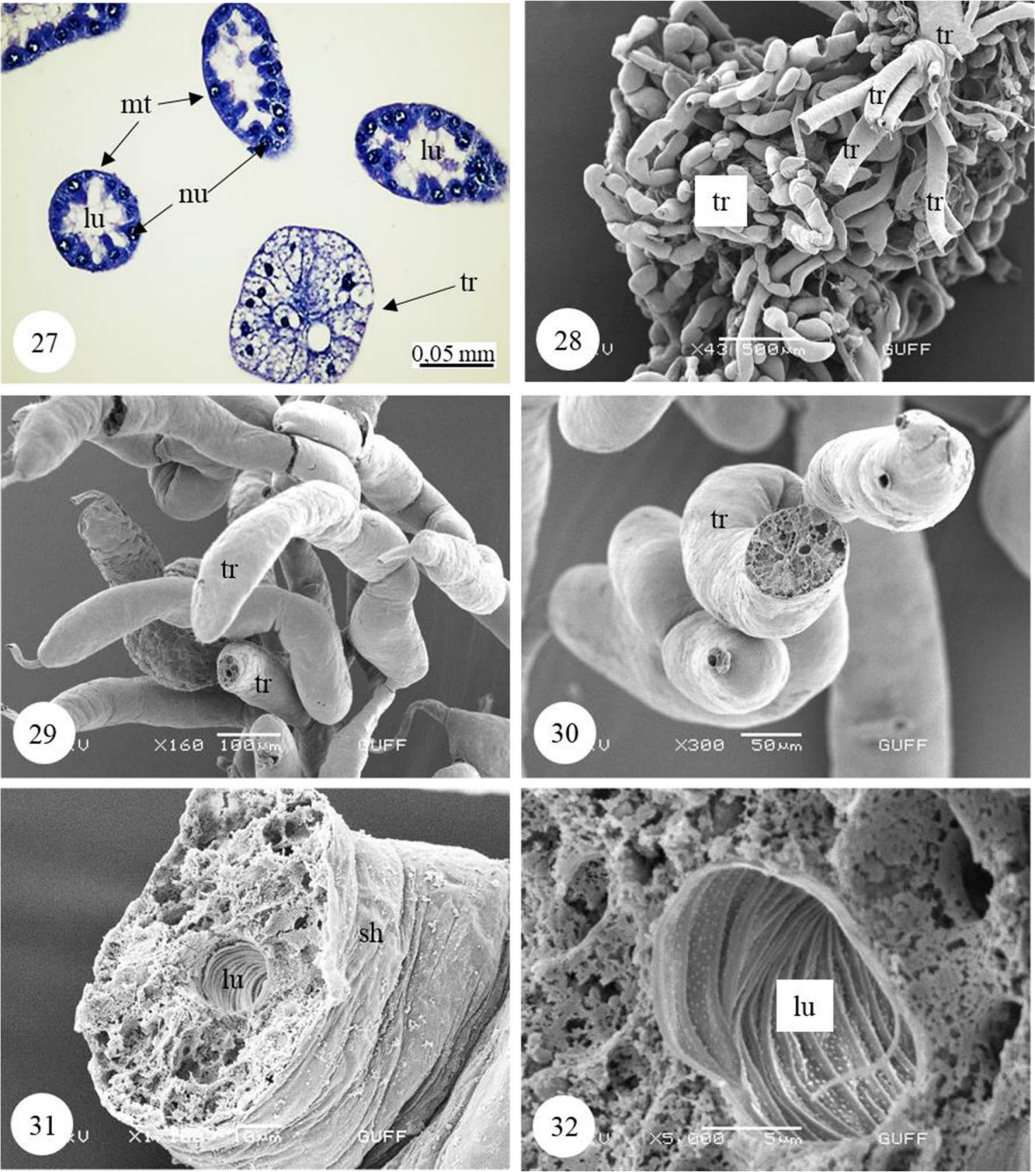
Cross-section of Malpighian tubule and trachea (H&E), (LM). **28–29**. General view of balloon-shaped trachea (SEM). **30–32**. SEM photographs of the transversely broken trachea. *Mt* Malpighian tubule, *tr* trachea, *lu* lumen, *nu* nucleus, *sh* sheath, *H&E* hematoxylin-eosin, *LM* light microscope, *SEM* scanning electron microscope

Malpighian tubules are two pairs, and their proximal ends are connected between the midgut and hindgut (Fig. 19). In the photographs of Malpighian tubules, it is distinguished that they form thin, long, twisted tubules (Figs. 21, 22). In histological sections of these tubules, a single-layered cuboidal epithelium with round nuclei is distinguished (Figs. 20, 27). Nuclei occupy almost the entire cell and are dense with chromatin (Fig. 27). Epithelial cells have brush border at their apical part (Figs. 20, 27). Tracheas with many balloon-like structures between Malpighian tubules are found (Fig. 22). Malpighian tubules have a flat surface (Fig. 23). In the cross sections of dried and transversely broken tubules, long microvilli extending from the apical of the single-layered cuboidal epithelium towards the lumen are observed (Figs. 24, 25, 26). Secretory granules were found in the lumen of Malpighian tubules (Fig. 26).

Many trachea are found in *C. felschei*, especially in the midgut and Malpighian tubule surfaces (Figs. 17, 22). It is seen that they are approximately the same thickness in the sections taken from the trachea and Malpighian tubules and in the SEM photographs (Figs. 23, 27). It has been distinguished that the tracheal structures in this species are quite different from other Coleopteran species; the trachea is in the form of long tubular structures, and these structures are divided into side branches by forming finger-shaped sacs (Figs. 28, 29, 30). It has been observed that the respiratory system has a more complex structure than other species (Figs. 28, 29, 30, 31, 32). In LM and SEM of the trachea, it is seen that it has a spongy structure with hollow chambers (Figs. 27, 31, 32). Many small protrusions in dots were found on the lumen surface (Fig. 32).

## Discussion

The Scarabaeidae family includes phytophagous and saprophagous species. Coprinae are saprophagous insects that feed on carrion, dung, decaying materials, and fungi (Ritcher 1958). In grazing ecosystems, dung-feeding beetles are known to play a significant role in nutrient cycling (Tixier et al. 2015).

Dung beetles are classified into four functional groups based on their nesting behaviors. The most significant are the tunnellers (paracoprids), which burrow into the soil beneath the dung pile, using it for both feeding and reproduction. The rollers (telecoprids) follow them, who roll dung into balls and transport them to different locations before feeding on them or laying their eggs inside. Another group consists of the dwellers (endocoprids), who lay their eggs directly within the dung and remain there to feed. Lastly, the kleptoparasites exploit the nests of tunnellers or rollers by laying their eggs in those nests (Holter and Scholtz 2005).

The genus Copris consists of paracoprid dung beetles, characterized by their behavior of tunneling beneath dung to bury it as a resource for feeding and reproduction. Copris are of the mandibulate type, specialized for gathering, masticating, and ingesting solid food (Miller 1961).

The anatomical and histological structure of the Coleoptera alimentary canal reveals differences that are correlated with their various feeding strategies. The three main regions of the *C. felschei *alimentary canal, known as the foregut, midgut, and hindgut, are easily distinguishable and are essentially similar to those in other Coleopterans. For example, the alimentary canal of *Diplotaxis liberta* (Scarabaeidae), *Prodontria lewisii* (Scarabaeidae), *Cephalodesmius armiger* (Scarabaeidae), *Hypothenemus hampei* (Curculionidae), *Epiphaneus malachiticus* (Curculionidae), Tanymecus dilaticollis (Curculionidae), Calosoma sycophanta (Carabidae), *Capnodis tenebrionis* (Buprestidae), and *Chrysolina herbacea* (Chrysomelidae) consists of three parts (Jones 1940; Ferreira and McKinlay 2001; Lopez-Guerrero 2002; Rubio et al. 2008; Candan et al. 2019, 2020a, 2020b; Özyurt Koçakoğlu et al. 2020a; Özyurt Koçakoğlu and Candan 2021a). However, the lengths of these three main parts and the parts they contain may vary interspersifically.

The midgut in *C. felschei*, *P. lewisii* (Coleoptera: Scarabaeidae), and *C. tenebrionis* (Buprestidae) are not divided into parts (Ferreira and McKinlay 2001; Özyurt Koçakoğlu et al. 2020a). However, the midgut of *D. liberta* (Scarabaeidae), *H. hampei* (Ferrari) (Curculionidae), *E. malachiticus* (Curculionidae), and *Chrysomela populi* (Chrysomelidae) are divided into parts (Jones 1940; Rubio et al. 2008; Candan et al. 2019; Özyurt Koçakoğlu et al. 2022b).

Comprising roughly one-third of the total digestive system, the midgut is the largest and most significant portion of the digestive tract, as considerable digestion occurs within this section. In *C. felschei*, the midgut is longer, while the foregut and hindgut are quite short. A similar situation is observed in *D. liberta* (Scarabaeidae), *P. lewisii* (Scarabaeidae), *C. armiger* (Scarabaeidae), and *H. hampei* (Curculionidae) (Jones 1940; Ferreira and McKinlay 2001; Lopez-Guerrero 2002; Rubio et al. 2008). In another study, the foregut and hindgut in *C. herbacea* (Chrysomelidae) are almost equal in length, but the midgut is longer than these parts (Özyurt Koçakoğlu and Candan 2021a).

The findings of this study reveal the morphological and functional diversity of midgut structures in various insect species. In *C. felschei*, the midgut is covered with a single-layered cylindrical epithelium consisting of long columnar cells continuously renewed by specialized regenerative cells, which are dispersed or form lateral crypts. A muscular layer and numerous tracheae also cover the surface. Additionally, a peritrophic membrane was identified in the midgut lumen. Similar structures in *C. sycophanta* (Carabidae) (Candan et al. 2020b) support general morphological trends regarding the role of the peritrophic membrane in covering food particles and the presence of crypt structures on the midgut surface. In *E. malachiticus* (Curculionidae), midgut epithelial cells are characterized by their elongated and cylindrical form, consisting of small epithelial cells with numerous tracheae and tracheoles on the midgut surface (Candan et al. 2019). Similarly, *D. liberta* (Scarabaeidae), *C. armiger* (Scarabaeidae), and *H. hampei* (Curculionidae) can be given as examples (Jones 1940; Lopez-Guerrero 2002; Rubio et al. 2008). In conclusion, the presence of peritrophic membranes reflects significant adaptations related to insect feeding strategies. The peritrophic membrane is found in insects that feed on solid food, preventing damage to epithelial cells. The data obtained from these studies contribute to a broader understanding of midgut structural variations among different insect species and their roles in food processing.

The tracheal structure may vary depending on oxygen needs in different species. In *C. felschei*, the trachea is a complex system of branched, long, tubular, annular canals connected to numerous, branched, large, lobed, bubble-shaped air sacs. But in *T. dilaticollis*, *C. tenebrionis*, *E. ovulum*, *M. cernyi*, and *C. populi*, the trachea was seen as simpler, thin, long tubes (Candan et al. 2020a; Özyurt Koçakoğlu et al. 2020a, 2020b; 2022a, 2022b). As a result, the tracheal system in *C. felschei* has a more complex structure than other compared species because this species, which feeds by digging tunnels in the dung, requires more oxygen (Candan et al. 2019, 2020a, 2020b; Özyurt Koçakoğlu et al. 2021a, 2021b, 2022a, 2022b).

Malpighian tubules can be freely distributed in the abdominal cavity, or their ends may connect to the colon, forming a cryptonephric system (Bradley 1985). In species of the Coleoptera order, the distal end of the Malpighian tubules is connected to the wall of the hindgut in accordance with the cryptonephric system. However, in different species of this order, it can sometimes be connected to the first (ileum) or second part (colon) of the hindgut, sometimes to the last part (rectum wall), sometimes to the colon and rectum wall, or the wall of the entire hindgut. In *C. felschei*, the distal ends of Malpighian tubules are connected between the ileum and colon. However, in *C. sycophanta* (Carabidae) and *C. herbacea* (Chrysomelidae), distal ends of Malpighian tubules surround the colon (Candan et al. 2020b; Özyurt Koçakoğlu and Candan 2021a).

Malpighian tubules show different color variations. Malpighian tubules maintain homeostasis by excreting various fluids and substances into the hemolymph and eliminating waste and toxic materials from the body. Consequently, color variations can be linked to these physiological processes (Özyurt Koçakoğlu et al. 2022a, b). In *C. felschei*, Malpighian tubules are transparent in color. In *Carabus violaceus* (Carabidae), they are creamy white. However, in *C. sycophanta* (Carabidae), Malpighian tubules are light orange (Ali 1964; Candan et al. 2020b). Additionally, the number of Malpighian tubules also varies by species. There are four Malpighian tubules in *C. felschei*. Similarly, there are four in *D. liberta* (Scarabaeidae), *C. armiger* (Scarabaeidae), and *C. populi* (Chrysomelidae). However, there are six in *H. hampei *(Curculionidae) and *T. dilaticollis* (Curculionidae) (Jones 1940; Lopez-Guerrero 2002; Candan et al. 2020a; Özyurt Koçakoğlu et al. 2022b).

This study is expected to contribute to a deeper understanding of the alimentary canal in Scarabaeidae. Additionally, the information acquired through this research will benefit future investigations seeking to advance knowledge of Scarabaeidae alimentary canal and their ecological roles.

## Data Availability

The data supporting this study’s findings are available in this article’s supplementary material.

## References

[CR1] Aldigail SA, Alsaggaff AI, Al-Azab AM (2013) Anatomical and histological study on the digestive canal of *Epilachna chrysomelina* (Coleoptera: Coccinellidae). Biosci Biotechnol Res Asia 10(1):183–192. 10.13005/bbra/1109

[CR2] Ali HA (1964) An introduction to the taxonomy of Iraqi Carabidae Col., examining the taxonomic value of internal characters. Imperial College of Science and Technology, Department of Zoology and Applied Entomology, South Kensington, London.

[CR3] Aslam NA (1961) An assessment of some internal characters in the higher classification of the Curculionidae S. L. (Coleoptera). Trans R Entomol Soc Lond 1113:417–480. 10.1111/j.1365-2311.1961.tb00799.x

[CR4] Bess HA (1935) The alimentary canal of *Calosoma sycophanta* Linnaeus. Ohio J Sc 35:54–61

[CR5] Borror DJ, Delong DM, Triplehorn CA (1976) An introduction to the study of Insects, 4th edn. Holt, Rinehart and Winston, New York

[CR6] Bradley TJ (1985) The excretory system: structure and physiology. Pergamon Press, New York

[CR7] Calder AA (1989) The alimentary canal and nervous system of Curculionoidea (Coleoptera): Gross morphology and systematic significance. J Nat Hist 23:1205–1265. 10.1080/00222938900770671

[CR8] Candan S, Özyurt Koçakoğlu N, Erbey M (2019) Morphology and histology of the alimentary canal of *Epiphaneus malachiticus* Boheman, 1842 (Coleoptera, Curculionidae). Entomol Rev 99:326–336. 10.1134/S0013873819030059

[CR9] Candan S, Özyurt Koçakoğlu N, Güllü M, Çağlar Ü (2020a) Anatomical and histological studies of the alimentary canal of adult maize leaf weevil, *Tanymecus dilaticollis* Gyllenhal, 1834 (Coleoptera: Curculionidae). Microsc Res Tech 83(9):1153–1162. 10.1002/jemt.23507 32483898 10.1002/jemt.23507

[CR10] Candan S, Özyurt Koçakoğlu N, Serttaş A (2020b) Histoanatomy of Malpighian tubules and the digestive tract of adult of biocontrol agent *Calosoma sycophanta* L. (Coleoptera: Carabidae). Int J Trop Insect Sci 41:1373–1386. 10.1007/s42690-020-00331-4

[CR11] Crome W (1957) Zur Morphologie und anatomie der larve von *Oryctes nasicornis* L. (Col. Dynastidae). Dtsch Entomol Z 4:254–257

[CR12] Crowson RA (1981) The biology of Coleoptera. Academic press, London, UK

[CR13] De Sousa G, Scudeler EL, Abrahão J, Conte H (2013) Functional morphology of the crop and proventriculus of *Sitophilus zeamais* (Coleoptera: Curculionidae). Ann Entomol Soc Am 106(6):846–852. 10.1603/AN13081

[CR14] Dow JAT (1986) Insect Midgut Function Adv Insect Physiol 19:187–328. 10.1016/S0065-2806(08)60102-2

[CR15] Ekis G, Gupta AP (1971) Digestive system of Cleridae (Coleoptera). Int J Insect Morphol Embryol 1:51–86. 10.1016/0020-7322(71)90008-0

[CR16] Ferreira SM, McKinlay B (2001) Gross features of the alimentary canal and reproduction organs *in Prodontria lewisii* (Coleoptera: Scarabaeidae: Melolonthinae). N Z J Zool 28(3):263–271. 10.1080/03014223.2001.9518269

[CR17] Gillott C (2005) Entomology, 3rd ed, Springer Science and Business Media, Dordrecht, The Netherlands.

[CR18] Grebennikov V, Scholtz C (2004) The basal phylogeny of Scarabaeoidea (Insecta: Coleoptera) inferred from larval morphology. Invertebr Syst 18:321–348. 10.1071/IS03013

[CR19] Halffter G, Halffter V (2009) Why and where coprophagous beetles (Coleoptera: Scarabaeinae) eat seeds, fruits or vegetable detritus. Boletín De La S E A 45:1–22

[CR20] Holter P, Scholtz CH (2005) Are ball-rolling (Scarabaeini, Gymnopleurini, Sisyphini) and tunnelling scarabaeine dung beetles equally choosy about the size of ingested dung particles? Ecol Entomol 30(6):700–705

[CR21] Jaspar-Versali MF, Goffinet G, Jeuniaux C (1987) The digestive system of adult carabid beetles: an ultrastructural and histoenzymological study. Acta Phytopath Entom Hung 22(1–4):375–382

[CR22] Johnson KS, Rabosky D (2000) Phylogenetic distribution of cysteine proteinases in beetles: evidence for an evolutionary shift to an alkaline digestive strategy in Cerambycidae. Comp Biochem Physiol Part B 126:609–619. 10.1016/S0305-0491(00)00232-7 10.1016/s0305-0491(00)00232-7 11026673

[CR23] Jones CR (1940) The alimentary canal of *Diplotaxis liberta* germ (Scarabaeidae: Coleoptera). Ohio J Sci 40(2):94–103

[CR24] Löbl I, Daniel L (2016) Catalogue of palaearctic Coleoptera: revised and updated edition Scarabaeoidea – Scirtoidea–Dascilloidea–Buprestoidea–Byrrhoidea. Vol. 3. 2nd ed, Boston, Brill Leiden.

[CR25] Lopez-Guerrero Y (2002) Anatomy and histology of the digestive system of *Cephalodesmius armiger* Westwood (Coleoptera, Scarabaeidae, Scarabaeinae). Coleopts Bull Journal 56(1):97–106. 10.1649/0010-065X(2002)056[0097:AAHOTD]2.0.CO;2

[CR26] Mehrabadi M, Bandani AR, Allahyari M, Serrão JE (2012) The Sunn pest, *Eurygaster integriceps* Puton (Hemiptera: Scutelleridae) digestive tract: histology, ultrastructure and its physiological significance. Micron 43(5):631–637. 10.1016/j.micron.2011.11.008 22227010 10.1016/j.micron.2011.11.008

[CR27] Miller A (1961) The mouth parts and digestive tract of adult dung beetles (Coleoptera: Scarabaeidae), with reference to the ingestion of helminth eggs. J Parasitol 47(5):735–744 14473936

[CR28] Nichols E, Spector S, Louzada J, Larsen T, Amezquita S, Favila ME (2008) Ecological functions and ecosystem services provided by Scarabaeinae dung beetles. Biol Conserv 141(6):1461–1474. 10.1016/j.biocon.2008.04.011

[CR29] Noriega JA, Calle JC (2008) Consumption of *Gustavia hexapetala* (Aublet) Smith (Lecythidales: Lecythidaceae) by the dung beetle *Eurysternus plebejus* Harold (Coleoptera: Scarabaeidae). Coleopt Bull 62:455–460. 10.1649/1091.1

[CR30] Özyurt Koçakoğlu N, Çağlar Ü, Candan S (2021b) Anatomy and histology of digestive tract in *Melanophila* (*Trachypteris*) *picta decastigma* (Fabricius, 1787) (Coleoptera: Buprestidae). Eur J Biol 80:1–8. 10.26650/EurJBiol.2021.800131

[CR31] Özyurt Koçakoğlu N, Candan S (2021a) Characterization of the alimentary canal and Malpighian tubules of *Chrysolina herbacea* (Duftschmid, 1825)(Coleoptera: Chrysomelidae): Anatomical and histological approaches. Microsc Res Tech 84(6):1135–1144. 10.1002/jemt.23671 10.1002/jemt.23671 33305860

[CR32] Özyurt Koçakoğlu N, Candan S, Çağlar Ü (2020a) Histomorphology of the adult digestive tract of *Capnodis tenebrionis* (L. 1758) (Coleoptera, Buprestidae). Microsc Microanal 26(6):1245–1254. 10.1017/S1431927620024472 10.1017/S1431927620024472 33161937

[CR33] Özyurt Koçakoğlu N, Candan S, Erbey M (2020b) Structure of the mouthparts and alimentary canal of *Eusomus ovulum* Germar, 1824 (Coleoptera: Curculionidae). Rev Bras Entomol 64:e20200004. 10.1590/1806-9665-RBENT-2020-0004

[CR34] Özyurt Koçakoğlu N, Candan S, Güllü M (2022a) Anatomical and histological descriptions of digestive canal and excretory system of *Mylabris cernyi* Pan & Bologna, 2014 (Coleoptera: Meloidae). Orient Insects 56:362–378. 10.1080/00305316.2021.1991853

[CR35] Özyurt Koçakoğlu N, Candan S, Güllü M (2022b) Anatomy and histology of digestive tract in the red poplar leaf beetle *Chrysomela populi* Linnaeus, 1758 (Coleoptera: Chrysomelidae). Int J Trop Insect Sci 42:927–939. 10.1007/s42690-021-00619-z

[CR36] Poll M (1932) Contribution à l’étude des tubes de Malpighi des Coléoptères: Leur utilité en phylogenèse. Recl L’institut Zool Torley-Rousseau 4:47–80

[CR37] Raine EH, Slade EM (2019) Dung beetle-mammal associations: methods, research trends and future directions. Proc Biol Sci 286(1897):2018–2002. 10.1098/rspb.2018.2002 10.1098/rspb.2018.2002 PMC6408906 30963853

[CR38] Ritcher PO (1958) Biology of Scarabaeidae. Annu Rev Entomol 3:311–334. 10.1146/annurev.en.03.010158.001523

[CR39] Romoser WS, Stoff olano JG, (1998) The science of entomology, 4th edn. McGraw-Hill, Boston

[CR40] Rubio GJD, Bustillo PAE, Vallejo ELF, Acuña ZJR, Benavides MP (2008) Alimentary canal and reproductive tract of *Hypothenemus hampei* (Ferrari)(Coleoptera: Curculionidae, Scolytinae). Neotrop Entomol 37:143–151. 10.1590/s1519-566x2008000200006 18506292 10.1590/s1519-566x2008000200006

[CR41] Saini SR (2009) Histology and physiology of the cryptonephridial system of insects. Trans R Entomol Soc Lond 116:347–392. 10.1111/j.1365-2311.1964.tb02302.x

[CR42] Scholtz CH, Davis ALV, Kryger U (2009) *Evolutionary biology and conservation of dung beetles*. Pensoft, Sofia, pp. 567.

[CR43] Sinha RN (1958) The alimentary canal of the adult of *Tribolium castaneum* Herbst (Coleoptera: Tenebrionidae). J Kans Entomol Soc 31(2):118–125

[CR44] Snodgrass RE (1993) Principles of insect morphology, 2nd edn. Cornell University Press, New York

[CR45] Song YQ, Dong JF, Sun HZ, Liu ST (2012) Anatomical structure of the digestive tract of *Holotrichia oblita* Fald (Coleoptera: Melolonthidae). J Hunan Agric Univ Nat Sci 38:511–514. 10.3724/SP.J.1238.2012.00511

[CR46] Stammer HJ (1934) Bau und bedeutung der malpighischen gefässe der Copeopteren. Z Morph Ökol Tiere 29:196–217

[CR47] Terra RW (1990) Evolution of digestive systems of insects. Annu Rev Entomol 35:181–200

[CR48] Thomas JB (1967) A comparative study of gastric caeca in adult and larval stages of bark beetles (Coleoptera: Scolytidae). Proc Entomol Soc Ont 97:71–90

[CR49] Tixier T, Bloor JMG, Lumaret JP (2015) Species-specific effects of dung beetle abundance on dung removal and leaf litter decomposition. Acta Oecol 69:31–34. 10.1016/j.actao.2015.08.003

[CR50] Toni ASB, Fialho VS, Cossolin JFS, Serrão JE (2022) Larval and adult digestive tract of the carrion beetle *Oxelytrum discicolle* (Brullé, 1840) (Coleoptera: Silphidae). Arthropod Struct Dev 71:101213. 10.1016/j.asd.2022.101213 36208618 10.1016/j.asd.2022.101213

[CR51] Wigglesworth VB (1972) The principles of ınsect physiology, 7th edn. John Wiley and Sons Inc, New York

[CR52] Yang QF, Li Q, Zhi YR (2009) Anatomical study of the alimentary canal of *Xylosandrus germanus*. Chin Bull Entomol 46:623–626

[CR53] Yang YH, Shi MW, Yuan L (2011) Function and microstructure observation of Malpighian tubules in *Tenebrio Molitor*. J Henan Inst Sci Technol Nat Sci Ed 39:30–32

[CR54] Ziani S (2017) Morphological revision of the Western Palaearctic Species of the Genus Copris Geoffroy, 1762 with Three Foretibial External Teeth (Coleoptera: Scarabaeoidea: Scarabaeidae). Insecta Mundi: A Journal of World Insect Systematics 0528:1–26

